# When epidemiological databases inform injury mechanisms: biomechanical analysis of injury associations

**DOI:** 10.1186/s12889-022-14889-w

**Published:** 2023-01-11

**Authors:** Claire Bruna-Rosso, Nadim Ballout, Pierre-Jean Arnoux, Amina Ndiaye, Jean-Louis Martin, Céline Vernet

**Affiliations:** 1 Laboratoire de Biomécanique Appliquée, Aix Marseille University, University Gustave Eiffel, Marseille, France; 2 Unité Mixte de Rercherche Epidémiologique et de Surveillance Transports, Travail, Environnement, University Gustave Eiffel, University Claude Bernard Lyon 1, Bron, France

**Keywords:** Injury associations, Car crash, Injury mechanisms

## Abstract

**Background:**

Vehicle accidents are still a heavy social burden despite improvements due the latest technologies and policies. To pursue the trend of decrease, having a more detailed view and understanding of the injury patterns would contribute to inform both the rescue team to optimize victim’s management and policymakers in order for them to tackle at best this issue.

**Methods:**

Two complementary analyses of injury associations were performed, one using a biomechanical classification and the other an anatomic one, computed on data stratified by car accident type (lateral or frontal). Our objective is to understand whether these two categories of crash lead to similar or heterogeneous injury association patterns, and analyze these findings from an impact mechanics point of view. Indeed, having an improved understanding of the injury mechanisms would facilitate their diagnosis and prevention.

**Results:**

While each type of accident possesses its own injury profile, most injury associations are found for both types. Injuries such as clavicle and rib fractures were identified as involved in a high number of associations. Several associations between fractures and blood vessel injuries were found.

**Conclusions:**

The results suggests three main conclusions: (i) Injury associations are rather independent from crash characteristics, (ii) Clavicle and rib fractures are typical of poly-traumatized victims, (iii) Certain fractures can be used to early detect victims at higher risk of hemorrhage. Overall, this study provide paramedics and doctors with data to orientate them toward a faster and more appropriate decision. Moreover, this exploratory work revealed the potential that injury association analyses have to inform policy-making and issue recommendations to decrease road accident mortality and morbidity.

## Background

Despite the latest advances in the field of road safety, the mortality and morbidity related to road accidents is still a major public health issue. Indeed, in the European Union (EU) more than 20000 peoples died and more that 1.4 million were injured in 2016 [1]. One way to reduce these numbers is to improve vehicles and infrastructure to make them safer. Another domain that can contribute to further lower the road accident sequels and deaths is to optimize the victims’ management. Indeed, time and triage accuracy are two key features that influence significantly the patient outcomes [2–5]. For both of these directions , an advanced understanding of the injuries suffered by the vehicle occupants and how they occur (i.e. the injury mechanisms) is fundamental. In fact, it can provide the rescue team with data to inform their decision-making process and/or contribute to orientate the development of passive safety devices. Automobile is the mode of transport the most frequently involved in serious and deathly accidents. Indeed, in the UE in 2016, car crashes were responsible for more than half of the road accident fatalities. Previous studies have investigated the relationship between the accident type, injuries and injury mechanisms [6–11]. They observed that lateral and frontal impacts are the most frequent crashes that lead to serious injuries. Theses studies also agreed on the fact that the two accident types lead to different lesion patterns, but they are rather simple in terms of methodology and only rely on counts and proportions. To the best of our knowledge, no studies have studied how the occurrence of one injury influences the presence of another with respect to the impact principal direction of force (PDOF). Recent studies have proposed a new method to analyze the injury profile from a stratified road-accident victim database, through the computation of multiple statistical injury associations [12]. Such a method allows to obtain the conditional probability (odd-ratio) of observing a given injury knowing that a specific one is present and to graphically represent these conditional dependencies between them. This method can be applied to stratified data, i.e. data separated according to predefined categories, such as type of road users, as illustrated in the cited article. This model can be used to retrieve injury profile and injury associations patterns with respect to the different strata. The present study proposes an investigation of the influence, in terms of injury mechanisms, of all the injury associations related to car-accident front passengers and drivers, according to the type of accident. A first section will introduce the database and the methods that were used within the scope of the study. It is followed by the results and a discussion of their implications in terms of road safety. A conclusion will then recall the main findings and the future works they suggest.

## Methods

### Epidemiological databases

This study relies on two data collections of road traffic casualties with complementary information:

- **The Rhône Registry of Road Traffic Casualties** Since 1995, all victims of road traffic crashes that occurred within the Rhône county administrative area and who benefited from medical care in public or private facilities are included in this registry. A more detailed description of the cohort protocol is available elsewhere [13]. The Rhône registry data collected in medical care units include a precise description of injuries as well as general information about crash circumstances and victims’ characteristics. This registry has been approved by the French National Registry Committee.
- **The French national police database of road traffic accidents** For each road crash involving at least one vehicle and with at least one injured casualty, French police forces are required to complete a police report. These reports are then compiled yearly to create a national crash database used for French road traffic crashes monitoring. This database includes for each victim rather precise information about the crash, vehicles and people involved. Contrary to the Rhône Registry, there is little information about injuries. More specifically for car crashes, it includes the accident type (frontal, lateral, rear, multiple crash events) and the location of the victim inside the car (driver, front or back passengers), information not available in the Rhône registry.

For the present study, data from these two databases were merged to obtain a complete database with a full description of injuries and car crash types. All methods were carried out in accordance with relevant guidelines and regulations in the “Ethics approval and consent to participate” section.

#### Injury classification

Two injury classifications were used:

- **The Abbreviated Injury Scale (AIS)** For a full description of injuries, the Abbreviated Injury Scale (AIS) 1990 was used [14], which is an internationally recognized traumatic injury scoring system. The AIS includes codes consisting of 6 digits that refer to body regions (R, first digit), types of anatomic structure (T, second digit), additional specifications of anatomic structure or location (S, third and fourth digits), and the specific type of lesion (N, fifth and sixth digits). For example, the code 440604 correspond to R: Thorax (4), S: Internal organs (4), T: Diaphragm (06) and N: Wound (rupture) (04), and therefore represents a wound or rupture injury in the diaphragm which is an internal organ in the thorax region. The Rhone registry merged with the French national police dataset contains 1348 distinct codes corresponding to 1348 distinct injuries. We will refer to these codes as the “RTSN” classification. The AIS includes also a severity metric, the “AIS score”, which is an ordinal scale, ranging from 1 (minor severity) to 6 (maximum severity, currently incurable). An additional score of 9 was introduced for injuries whose severity was not specified. The whole classification that includes the severity score is referred as the “RTSN+AIS” classification.
- **Custom-made “Biomechanical Classification” (BC)** The coding used in the BC is presented in Table 1. It consists in two numbers that respectively represent the body region (similarly to the RTSN classification), the type of tissue. It was conceived to regroup injuries sharing similar tissue characteristics, such as fractures or internal organ wounds within the same body region. We refer to this classification as “BC”. The AIS severity score was then also added, giving a classification that we refer to as “BC+AIS”. For instance, the code 5.3.4 represents a vein or artery injury in the abdominal region with an AIS4 severity.

The “Major internal organs” category includes:

- Head: Brain, cerebellum
- Thorax: Heart, diaphragm, lungs
- Abdomen: Liver, pancreas, kidney, small and large bowel, spleen, mesentery, stomach

The “Other internal organs” category includes:

- Face: internal ear, eye
- Neck: thyroid gland, vocal cords, pharynx, larynx, salivary gland
- Thorax: bronchi, esophagus, pericardium, trachea
- Abdomen: Suprarenal gland, anus, ovary, scrotum, penis, perineum, testicle, vagina, vulva

**Table 1:** Biomechanical classification coding for injuries

Code	Body region	Tissue / body structure	Severity
1	Head	Skin	Minor
2	Face	Nerves & Spinal cord	Moderate
3	Neck	Arteries and veins	Serious
4	Thorax	Muscles, tendons & ligaments	Severe
5	Abdomen	Bones & inter-vertebral discs (in spine)	Critical
6	Spine	Major internal organs	Maximal
7	Upper extremity	Loss of consciousness	
8	Lower extremity	Other internal organ	
9	Unspecified	Massive destruction	

Loss of consciousness and massive destruction cannot be directly linked to a particular body structure or tissue. However, these injuries were kept in the BC since it was derived from the AIS coding, under the codes 7.X.X and 9.X.X, respectively.

### Statistical methods

#### Data stratification

Previous studies have shown that different impact principal directions of force (PDOF) lead to different injury mechanisms, patterns and severity [6]. As a consequence, the assumption was made that the injury associations consecutive to each type of accident will as well be different. Stratification of data is made in order to divide the data into clusters that are hypothesized to display a certain homogeneity within the strata and heterogeneity with respect to the others. Consequently, we stratified the data according to four types of accident present in the Police Road Crash database: frontal, lateral, rear, and multiple crash events. Multiple crash events were removed because of a too small sample size available, leading to a weak statistical power to evidence injury associations. Rear impacts were not studied because they are most of the time less serious than the other type of crash [15], and are thus of least interest from a road safety point of view. As a result, only two PDOF observed in car crashes were exploited, namely frontal and lateral.

#### Data selection

In the merged database, a different version of the AIS was used for 2015 and subsequent years. In order to keep the largest sample size with homogeneous injury coding for frontal and lateral comparison, only casualties of 2014 and before were kept. We then selected all the victims of car crashes, positioned in the front row (passengers and drivers) and whose type of accident is known. This led us to work with 15,658 observations (13,097 and 2561 observations in the frontal and lateral stratum respectively).

#### Association computations

Each injury [resp. group of injury] can be modeled by a binary random variable which is set to 1 if the victim suffers from this injury [resp. from at least one injury in this group of injury] and 0 otherwise. Groups of injuries are considered as injuries in the rest of paper. Therefore, the injury table of a victim can be modeled as a realization of the random variable $\textbf{U}=(U_1,...,U_p)^T \in \{0,1\}^p$ where $U_j$ is a binary variable that indicates the presence of the injury *j* in the considered injury table and *p* is the cardinality of the set of all possible injuries. Using the RTSN classification, 634 injuries appear in at least one injury table, while 230 were identified in at least 10 victims. In the remainder of the study, only the injuries having a prevalence greater than 10/20110 were retained. Tables 2 and 3 summarize the number of injuries for each classification as well as the number of observations found in each stratum.

**Table 2:** Number of observations within the different strata

	Initial	Freq > 10	
		BC	RTSN
All	20110	20089	20016
Lateral	2561	2558	2550
Frontal	13097	13087	13033
Back	3818	3813	3806
Multiple	634	631	627

**Table 3:** Number of injuries within the different classifications

Classification	Initial	Freq > 10
RTSN	634	230
BC	43	35
BC+AIS	130	74

In this study, we will use graphical models as a tool to visually represent the associations that exist between injuries. The estimation of the injuries associations can be reduced to describing the joint distribution of the *p* binary variables. For this purpose, we applied a quadratic exponential binary model, or Ising model, which has been widely used in this context [16–19]. Each Ising model can be translated into a graph $\mathcal {G} =(V,E)$ where *V* is the set of *p* vertices corresponding to the *p* injuries and *E* is the set of edges that describe the conditional independence relationships among injuries. The edge is absent when $U_j$ and $U_\ell$ are independent conditionally on the other variables. More specifically, an edge is present between two injuries $(j,\ell )$ if and only if $OR_{j,\ell }\ne 1$, where $OR_{j,\ell }$ corresponds to the conditional odds-ratio of the association between the two injuries.

In the present study, we study the associations between injuries according to the type of accident. We therefore have *K* models to estimate, where *K* is the number of accident types. In our context, the estimations of these models require a method capable to make the selection of variables (for each model we have a $p(p + 1)/2$ parameters to be estimated) and at the same time to take into account the homogeneity that may exist between strata without masking the heterogeneities. In order to handle this, we used in this study the DataShared-SepLogit method, that has been shown to be very appropriate to deal with stratified data and with the best compromise between performance and computational time [12]. This method is based on the combination of the SepLogit [20] method and the DataShared lasso penalty. The first consists in estimating the parameters of the Ising model via *p* logistic regressions, while the second developed by [21] and [22], is used within the context of stratified regression. See [12] for more details.

## Results

### General results

**Fig. 1:**
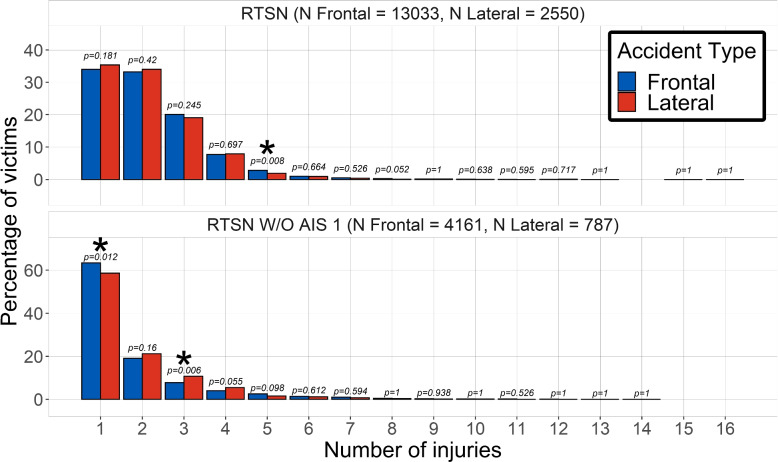
Distribution of the number of injury among car accident victims for lateral and frontal impact. Top: all AIS, bottom: AIS2+. Asterisks denote a statistical difference between lateral and frontal impacts (*p* < 0.05)

The number of injuries sustained by the victims for each strata is displayed in Fig. 1. This graphic shows that there is almost no statistical difference between front and lateral impacts from a simple prevalence point of view. After excluding the least severe injuries, there were more single injuries in frontal impacts compared to lateral impacts with more polytraumatized victims.

**Fig. 2:**
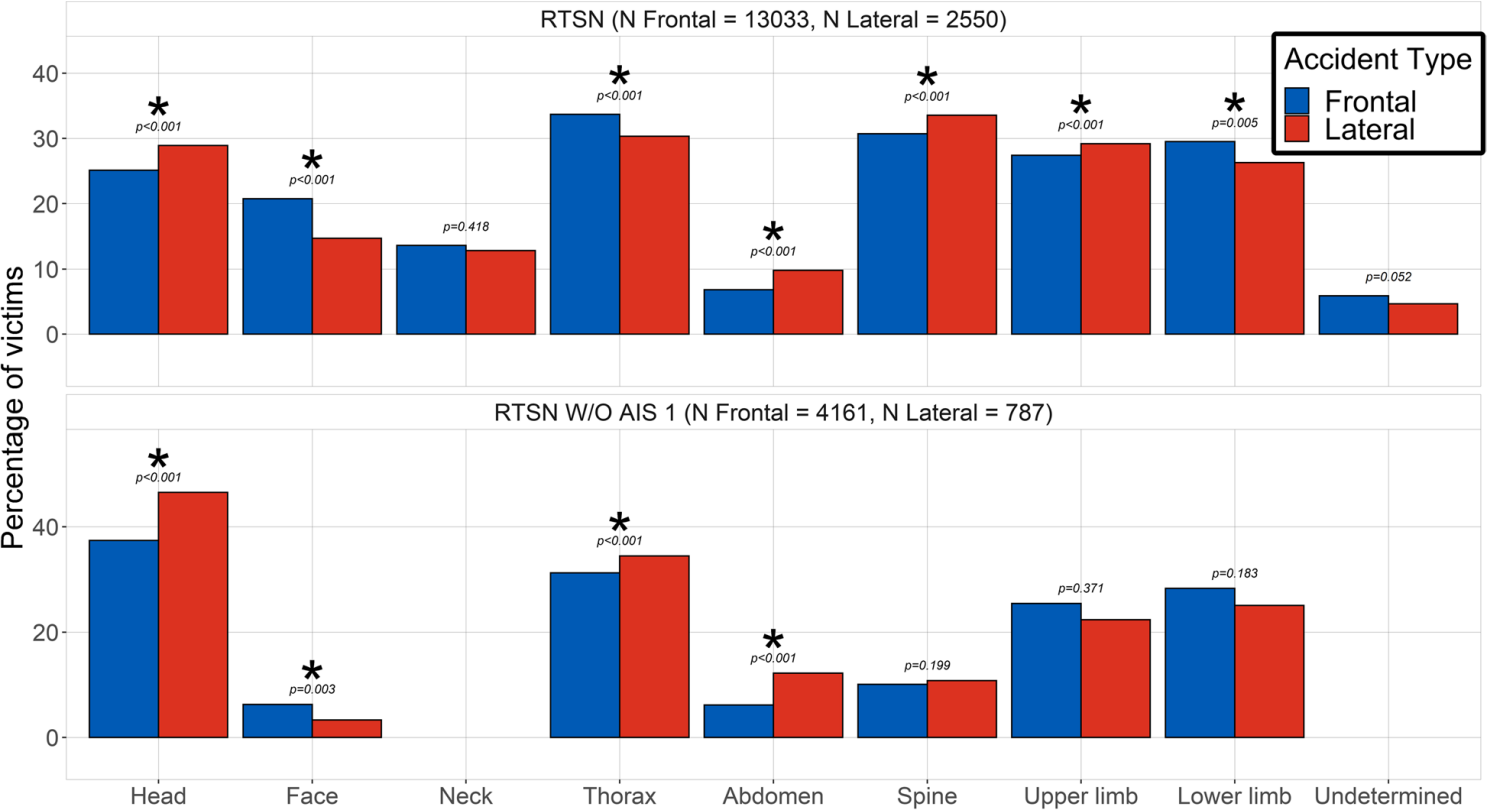
Distribution of the injuries by body region and accident type

The prevalence of injuries with respect to each body region is displayed in Fig. 2. In addition, the ten AIS2+ injuries coded using the BC with the highest prevalence for the frontal and lateral impacts are detailed in Table 4, in order to have an overview of the most frequent serious lesions suffered by car accident victims. In both lateral and frontal impacts, loss of consciousness is the most frequent AIS2+ injury, which is in accordance with various prior epidemiological studies [23, 24] that identified the head as the body region most frequently wounded in car accidents. In addition, the Fig. 2 reveals several particularities between the two accident types, which can be ascribed to probable corresponding injury mechanisms:

- More face trauma in frontal impact (20.73% vs. 14.71%, p<0.001). This can be caused by the direct contact between the victim face and the steering wheel or the airbag [25].
- More lower limb injuries in frontal impact (29.49% vs. 26.27%, p=0.005). This is attributable to the intrusion of the motor inside the driver compartment [26].
- More head injuries (25.09% vs. 28.9%, p<0.001), especially AIS2+ ones, in lateral impacts. This is most probably due to the reduced distance between the occupant and the door which leads to more severe head trauma, as well as coup/counter-coup injuries [9].
- More abdominal injuries in lateral impacts (6.81% vs. 9.8%, p<0.001). This might be due to the direct contact of the abdomen with the door armrest as well as compression of solids organs between the intruding objects and the spine [27]. This mechanism involves mostly the liver and the spleen, which are respectively wounded in 32 and 30 % of all abdominal traumas within the data used in the present study.

**Table 4:** Prevalence of the AIS2+ injuries labeled using the BC for frontal and lateral impacts

Frontal impact			Lateral impact		
Label	Prevalence	Body region & tissue type	Label	Prevalence	Body region & tissue type
1.7.2	0.09338	Head - Loss of consciousness	1.7.2	0.10164	Head - Loss of consciousness
4.5.2	0.05914	Thorax - Bones	7.5.2	0.04887	Upper limb - Bones
7.5.2	0.05761	Upper limb - Bones	8.5.2	0.04261	Lower limb - Bones
8.5.2	0.5662	Lower limb - Bones	4.5.2	0.03948	Thorax - Bones
8.5.3	0.03706	Lower limb - Bones	8.5.3	0.03245	Lower limb - Bones
6.5.2	0.02843	Spine - Bones	4.6.3	0.02697	Thorax - Major internal organs
7.5.3	0.02292	Upper limb - Bones	6.5.2	0.02697	Spine - Bones
4.6.3	0.0188	Thorax - Major internal organs	4.5.3	0.02111	Thorax - Bones
4.5.3	0.01719	Thorax - Bones	5.6.2	0.02072	Abdomen - Major internal organs
8.4.2	0.01498	Lower limb - Muscles	7.5.3	0.01603	Upper limb - Bones

**Fig. 3:**
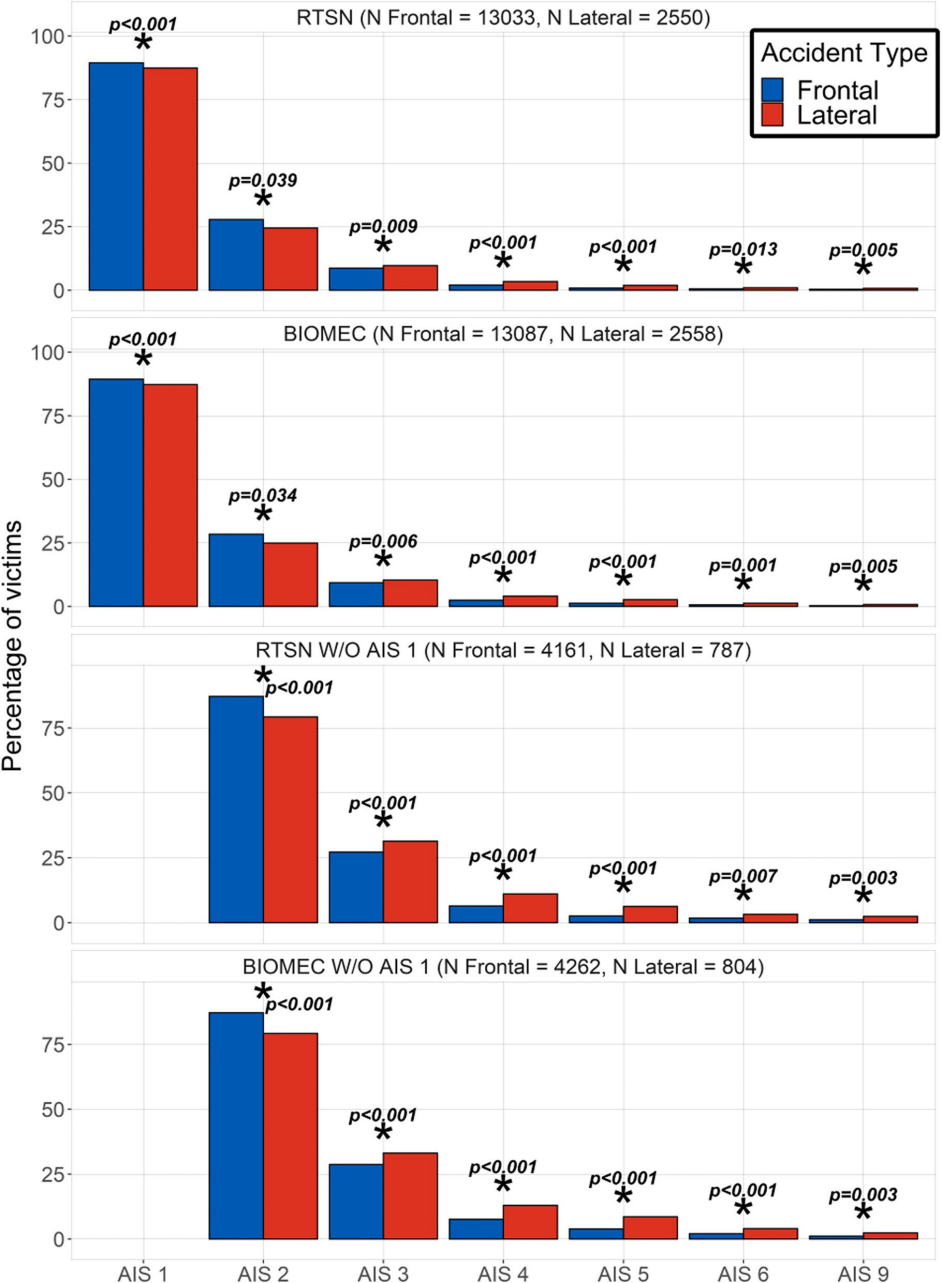
Distribution of the AIS severity for each accident type. a) All AIS b) without AIS1

The prevalence of each AIS severity for each type of accident is displayed in Fig. 3. The p-values that are reported in this figure were computed using a $\chi ^2$ comparison test between the proportions of victims with at least one injury within the AIS category vs. not. In accordance with previous studies (see [8] for instance), injuries are more severe for lateral impacts compared to frontal ones. Two motives might explain most of the difference in severity distribution between the two types of accident:

- Cars have a better ability to deform and thus absorb energy in frontal crashes compared with lateral ones [28];
- The human body is capable to sustain more severe solicitations in frontal rather than lateral direction [29].

### Injury associations using the BC

**Fig. 4:**
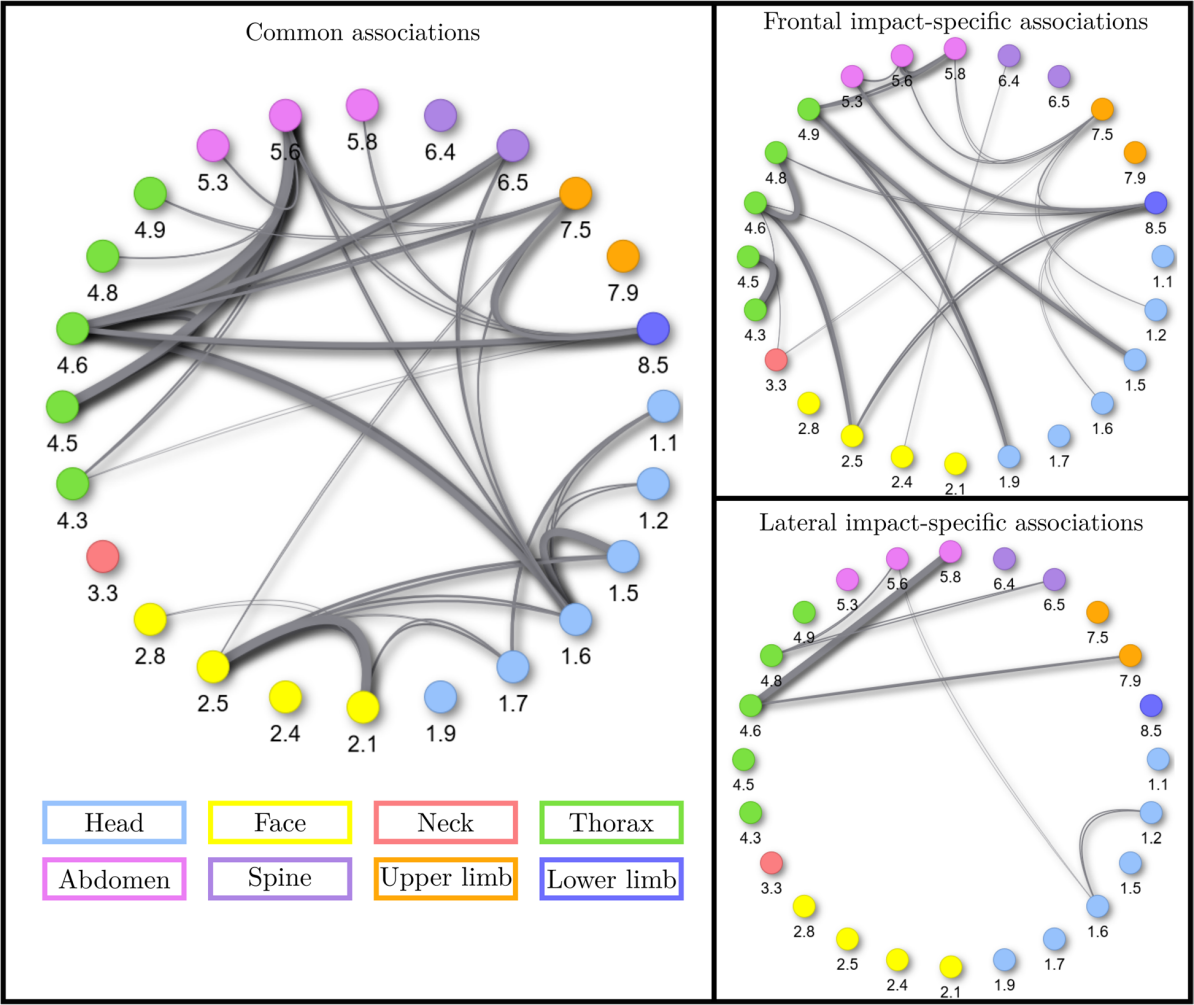
Injury associations using the BC

To get an overview of how the injured body regions and wounded structures are related to each other, the injury gravity was not considered in the first place. In a screening perspective to link epidemiological data to potential injury mechanisms, the BC is particularly fitted since it allows to group injuries that affect the same anatomical structure within a body region. A graphical representation of all the associations between two injuries that were computed for polytraumatized victims (*N* = 20089) is provided in Fig. 4. In that figure, each node corresponds to an injury coded using the BC, and each branch symbolizes an association between the two nodes. The branch width is representative of the association strength : the wider the branch, the higher the odd ratio of the associations between two injuries.

The diagram on the left hand side shows the common associations between lateral and frontal impacts. The two diagrams on the right hand side display the associations that were statistically significant specifically for the frontal (top) and lateral (bottom) impacts, apart from the common associations shown on the left hand side of the figure. In the frontal case, a total of 50 injury associations were found against 34 for the lateral one. This may be due to fewer lateral cases versus frontal ones in our dataset, and thus less statistical power to highlight injury associations. Among these associations, 19 are specific to frontal impacts and 3 to lateral impacts. 31 associations are common to both strata,so most of the associations are common to the two types of crash. A larger number of crash-specific association is found in the frontal case compared to the lateral case, most probably due to a higher number of cases available and consequently a higher statistical power. Overall, Fig. 4 shows that the injury association pattern is quite similar between frontal and lateral impacts. In both frontal and lateral crashes, the most represented body regions are head and thorax, which are as well the body regions in which the association density is the highest. Besides, neck, spine and limbs are less represented with respect to the other body regions in the two types of accident. The four strongest common associations include 4.6-5.6 (thorax internal organs/abdo internal organs) and 4.5-5.6 (thorax bones/abdo internal organs). They can be explained by the restrain system that compress both the rib cage and the abdominal region [30]. The other two strongest injury associations are 2.1-2.5 (face skin/bones) and 1.5-1.6 (head bones/organs). They link structures pertaining to the same body region, and are consequently rather expected. Indeed, it is foreseeable to have skin wounds related to bone wounds in the face, especially considering that this body part is seldom covered by clothes. Similarly, it is expected that cranial fractures (1.5) would lead to brain injuries (1.6). The strongest frontal impact-specific associations include 4.3-4.5 thorax arteries veins/bones, 4.6-4.8 thorax internal organs/other organs, 5.6-5.8 abdomen internal organs/other organs. The first is interesting since it reveals that an easy-to-diagnose wound (fracture) is characteristic of a life-threatening and more difficult to detect injury (thoracic hemorrhage). The last two associations show that frontal impact victims are at higher risk of having multiple internal organ injuries in the same body region. This is particularly interesting considering that the strongest lateral impact-specific association is between abdominal and thoracic internal organ injuries (4.6–5.8). This may orientate doctors in their diagnosis process toward paying more attention to organs located in the same body region of a previously identified injury in frontal impact victims, while in lateral impact a greater focus should be made on organs pertaining to different body regions.

### Injury associations using the BC+AIS classification

**Fig. 5:**
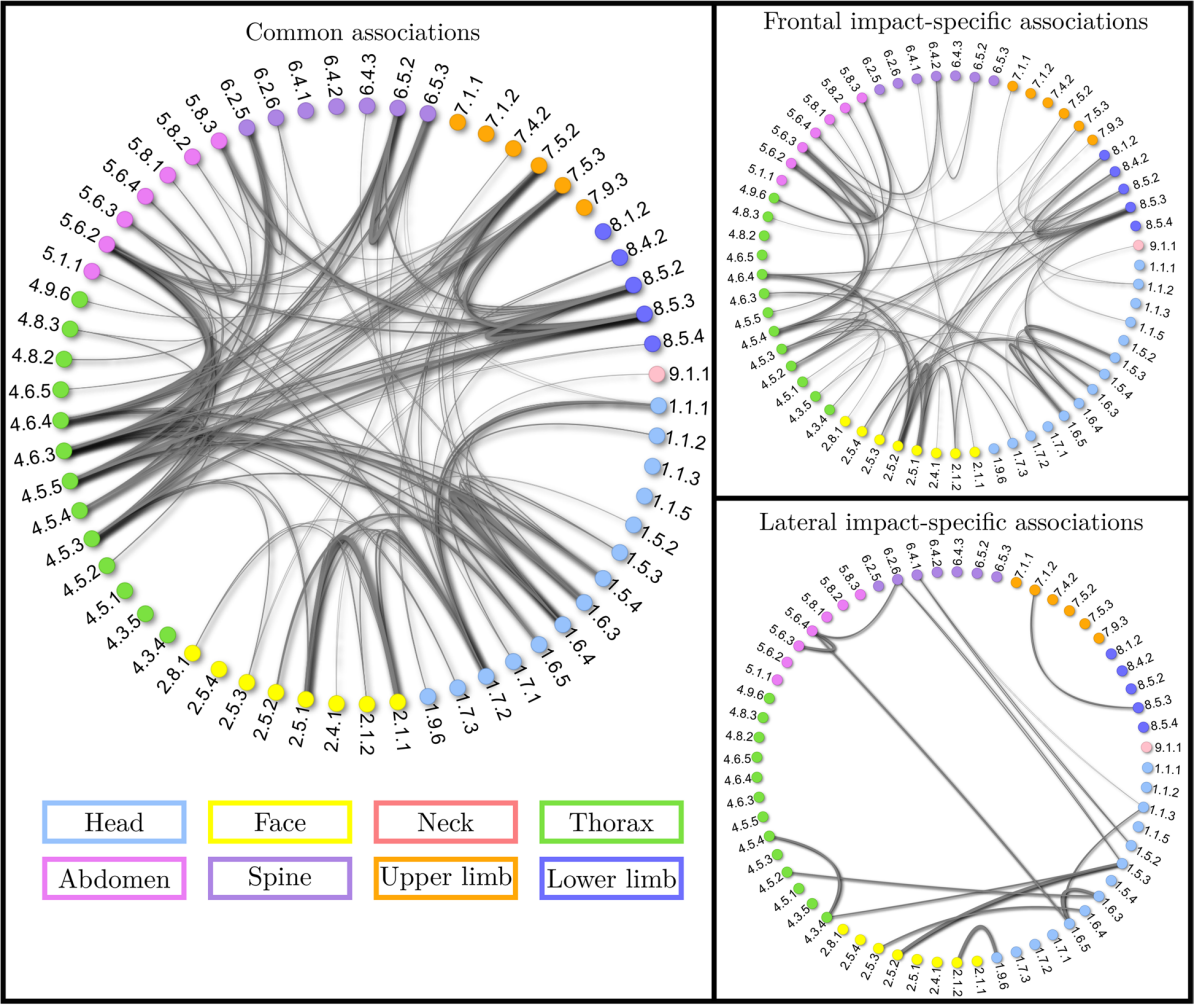
Injury associations using the BC+AIS

To get a more detailed overview of the injury associations, severity was included in the computations. The associations obtained using the BC+AIS classification are displayed in Fig. 5. As seen for BC without severity (Fig. 4), a majority of injury associations are located on the left-hand-side scheme and are thus common to both frontal and lateral crashes, resulting in mostly similar injury association patterns between frontal and lateral crashes. This result suggests that, for car-occupants, most of the associations between injuries are independent from the injury mechanism, approximated by the impact type (frontal/lateral). This means that the PDOF of the impact is unlikely to explain the simultaneous presence of two given injuries in a polytraumatized victim, except a few specific associations (shown on the right hand side panels of Figs. 4 and 5).

**Fig. 6:**
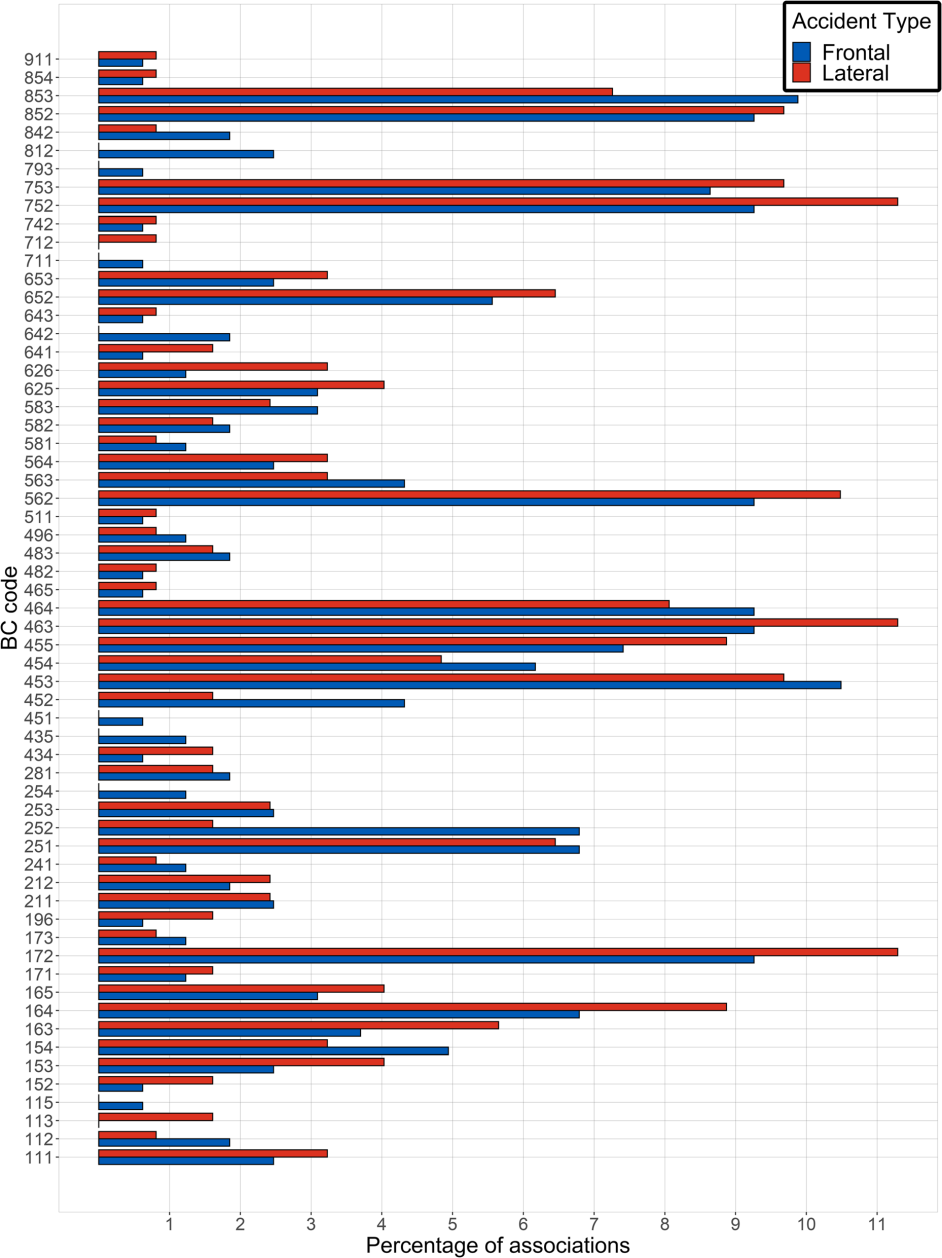
Distribution of the injury associations

The Fig. 6 displays the proportions of association in which each BC code is involved. It represents the number of branch that starts from each node of Fig. 5 divided by the total number of associations for each accident type (162 in the frontal case and 124 in the lateral case). For instance, the code 1.7.2 (head, loss of consciousness, AIS 2), with approximately the same prevalence in lateral and frontal crashes (Table 4), is involved in about 9.2% of all injury associations in frontal impact and in nearly 11.5% in lateral impact. The BC codes that are the most frequently involved in injury associations are gathered in Table 5. These lesions, by being the most frequently implicated in associations, are thus characteristics of polytraumatized car-accident victims. This is further confirmed by the fact that in this table several injuries appear more than once with different AIS severity codes (e.g. 4.6.3 and 4.6.4). Consequently, it can provide to the paramedics and doctors a supplementary information to choose the most appropriate procedure to take care of them.

**Table 5:** BC codes most frequently involved in injury associations

Frontal impact			Lateral impact		
Label	Frequency	Body region & tissue type	Label	Frequency	Body region & tissue type
4.5.3	17 (10.5%)	Thorax - Bone	1.7.2	14 (11.3%)	Head - Loss of consciousness
8.5.3	16 (9.9%)	Lower limb - Bone	4.6.3	14 (11.3%)	Thorax - Major internal organs
1.7.2	15 (9.3%)	Head - Loss of consciousness	7.5.2	14 (11.3%)	Upper limb - Bones
4.6.3	15 (9.3%)	Thorax - Major internal organs	5.6.2	13 (10.5%)	Major internal organs
4.6.4	15 (9.3%)	Thorax - Major internal organs	4.5.3	12 (9.7%)	Thorax - Bones
5.6.2	15 (9.3%)	Abdomen - Major internal organs	7.5.3	12 (9.7%)	Upper limb - Bones
7.5.2	15 (9.3%)	Upper limb - Bones	8.5.2	12 (9.7%)	Lower limb - Bone
8.5.2	15 (9.3%)	Lower limb - Bone	1.6.4	11 (8.9%)	Head - Major internal organs
7.5.3	14 (8.6%)	Upper limb - Bones	4.5.5	11 (8.9%)	Thorax - Bones
4.5.5	12 (7.4%)	Thorax - Bones	4.6.4	10 (8.1%)	Thorax - Major internal organs

### Injury associations using the RTSN classification

The listing of the RTSN codes of the injuries and their description that are most frequently involved in associations can be found in Tables 6 and 7. The graphs of associations obtained using the RTSN classification are provided in Fig. 8. Similarly to results using the BC+AIS (Fig. 5), most RTSN injury associations are common to the two impact orientations, and a larger number of impact-specific associations is found in the frontal case.

**Table 6:** RTSN codes most frequently involved in injury associations - Frontal impact

Code	Frequency	Label
450232	17 (14.7%)	Rib fractures with hemo- or pneumothorax, severe
210600	16 (13.8%)	Skin/subcutaneous/muscle laceration NFS^a^, minor
752200	12 (10.3%)	Clavicle fracture, moderate
450220	9 (7.8%)	Rib fractures, moderate
852604	9 (7.8%)	Open pelvic ring fracture NFS, severe
441410	8 (6.9%)	Bilateral lung contusion, severe
450804	7 (6.0%)	Sternum fracture, NFS, moderate

^a^*NFS*: No Further Specification

## Discussion

The major part of the injury associations that were computed can be classified as “obvious”, i.e. intuitively expected. For example, it is foreseeable that a cranial fracture (1.5.X) is associated with a brain injury (1.6.X). More generally, it is the case when different structures or injury types of the same body region are associated.

**Fig. 7:**
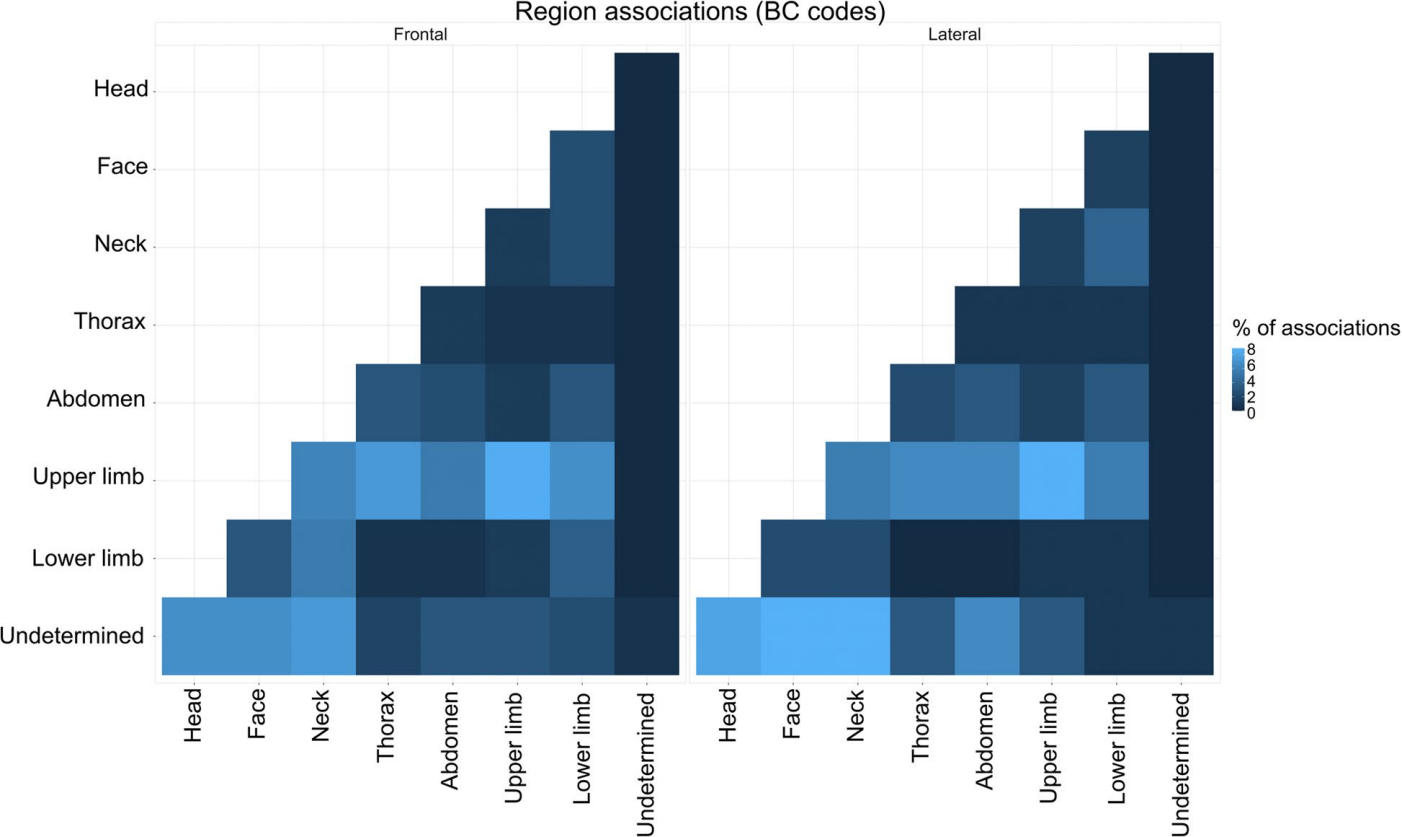
Distribution of the injury associations by body region

The heatmap Fig. 7 shows how the injury associations are distributed among body regions. For instance, in both frontal and lateral impact, close to 9% of all associations are between thorax and upper limbs. This figure suggests that during the diagnosis process in case of polytrauma, the investigations should not be restrained to the area were fractures are identified on the full body CT-scan, especially for the head and the thorax, for both accident types. While the distributions of associations mostly look alike between frontal and lateral impacts, several differences can be identified. Some of them can be explained from an impact dynamics point of view. For example, thorax and face (4,2) count more associations in frontal than in lateral impact. This could be due to the contact with the driving wheel (driver) or dashboard (front passengers). The same dynamics can explain the more frequent associations between the face and the lower limb (2,8) in frontal than lateral crash. Other discrepancies could be explained by the differences in mechanical properties of the human body in frontal and lateral directions. For instance, the higher number of associations between head and spine in lateral impact can be explained by the lower mobility and tolerance to impact of the spine in lateral direction. A mild shock or movement of the head sideways will thus lead to spine injury while similar ones along an antero-posterior axis would not [31].

The associations are also in accordance with clinically-observed phenomena. For instance, rib bone fractures (4.5.X) were identified in the literature as a good indicator of the seriousness of a victim [32]. The injury associations that were observed are consistent with these findings. In frontal impacts, and at a lower scale in lateral impacts, rib bone fractures are very frequently associated with other serious injuries. This result complements the finding reported in [32], which was based on prevalence only, with statistical associations. Moreover, our results allow to identify the body region, anatomical structure and severity of the injuries with which the rib bone fractures are associated and how strong is the association. This finding can as well be explained from a biomechanics point of view. Indeed, the rib cage acts as a protection to all the major internal organs contained in both the thorax and abdomen. Once this protective shield is damaged, it is very likely that underlying organs would be hurt as well. Moreover, the broken ribs might interfere with these organs, principally lungs, and generate lesions such as pneumo- or hemothorax.

By regrouping several AIS injury codes, the BC and BC+AIS allowed for a better statistical power with respect to the RTSN classification for certain kinds of injury. For instance, lower leg fractures are divided in several AIS injury codes and therefore RTSN injuries while gathered under the same codes in the BC. It turned out it is one of the most involved in injury association in frontal impact. This result could be explainable from an impact dynamic and biomechanical point of view. Indeed, lower limb injuries are a sign of high energy crash, because they are often due to the instrument panel intrusion in the driver and passenger environment that only occurs in high kinetic energy conditions in recent cars [33]. At lower speed, these intrusions are minor thanks to the motor unit compression which can absorb most of the kinetic energy of the crash. Lower limb injuries thus indicate a high-energy impact and consequently an increased risk of associated serious wounds, which agrees with our results. Moreover, they are typical of a polytraumatized patient and thus, can be used to identify a potentially seriously wounded victim in a car crash. In fact, it is well known that multiple injuries put patients at a higher risk than isolated wounds, which led to the utilization of indices such as the Injury Severity Score (ISS) [34].

The BC+AIS also made possible the identification of injuries that are related to a higher risk of hemorrhage, i.e. that are associated with veins/arteries wounds. Indeed, in the same way than lower limb fractures, by regrouping several AIS, the prevalence and statistical power associated to the BC+AIS codes relative to such wounds is high enough to retrieve associations. All blood vessel injuries that are involved in significant associations are localized in the thorax (code 4.3.X), and are related to bone fractures:

- Frontal case : Upper limb bone fracture AIS 3 (7.5.3) Head bone fracture AIS 3 and 4 (1.5.3-1.5.4) Thoracic bone fracture AIS 3 (4.5.3)
- Lateral case : Head bone fracture AIS 3 (1.5.3) Thoracic bone fracture AIS 3 (4.5.3)

Blood vessels injuries are key in terms of victims’ management. Indeed, they imply a high level of emergency since excessive blood loss is a life-threatening condition. For rescue and medical teams, it is capital to be able to diagnose them as soon as possible in order to make the appropriate decision concerning the procedure to handle the victim. The BC+AIS injury associations between vessels and bones can contribute to inform the decision-making process. Indeed, being easier to diagnose, the fractures previously listed can orientate the rescue team and doctors concerning the dedicated investigations to perform in order to detect the potential hemorrhage.

Together with associations that can be easily interpreted from well-known car crash injury mechanisms, less intuitive ones can be identified. For instance, upper limb fractures (7.5.X) are among the most frequently involved in injury associations. In order to analyze this finding, it is necessary to look closer into the details of the injury, which can be done using the RTSN classification. When studying the RTSN codes, in turns out that the specific upper limb wound which is the most frequently involved in injury associations is the clavicle fracture (AIS code 752200, see Tables 6 and 7). Unlike the rib fractures, where the broken ribs are known to interact with surrounding organs [32], notably the lungs, the associations between clavicle fractures and other injuries are probably not related to a direct relationship between the two wounds, i.e. the first in not the cause of the second. Indeed, in both frontal and lateral impacts, clavicle fractures are associated with thoracic injuries (rib bone fractures and lung contusions), brain injuries and pelvic fractures. This result suggests that, in the same way that lower limb fractures in frontal impact are characteristic of a high-energy crash, the clavicle fractures most probably translate a violent impact. Indeed, in frontal impact, they might be due to the the safety belt that, despite the load-limiter present in most of recent vehicles, gives rise to injuries. In lateral crash, they are due to direct impact against either the B-pillar or the striking object [35]. This injury thus appears as a good index to be used to inform triage decisions since it is frequently present in cases of polytrauma. This complies with previous clinical studies that pointed out clavicle fracture as a marker of serious polytrauma that can be used to indicate further investigations to diagnose other potential lesions [36, 37].

**Table 7:** RTSN codes most frequently involved in injury associations - Lateral impact

Code	Frequency	Label
210600	14 (17.9%)	Skin/subcutaneous/muscle laceration NFS, minor
450232	12 (14.1%)	Rib fractures with hemo- or pneumothorax, severe
752200	10 (12.8%)	Clavicle fracture, moderate
441410	9 (11.5%)	Bilateral lung contusion, severe
450220	7 (9.0%)	Rib fractures, moderate
210602	6 (7.7%)	Skin/subcutaneous/muscle laceration, superficial, minor
852604	6 (7.7%)	Open pelvic ring fracture NFS, severe

**Fig. 8:**
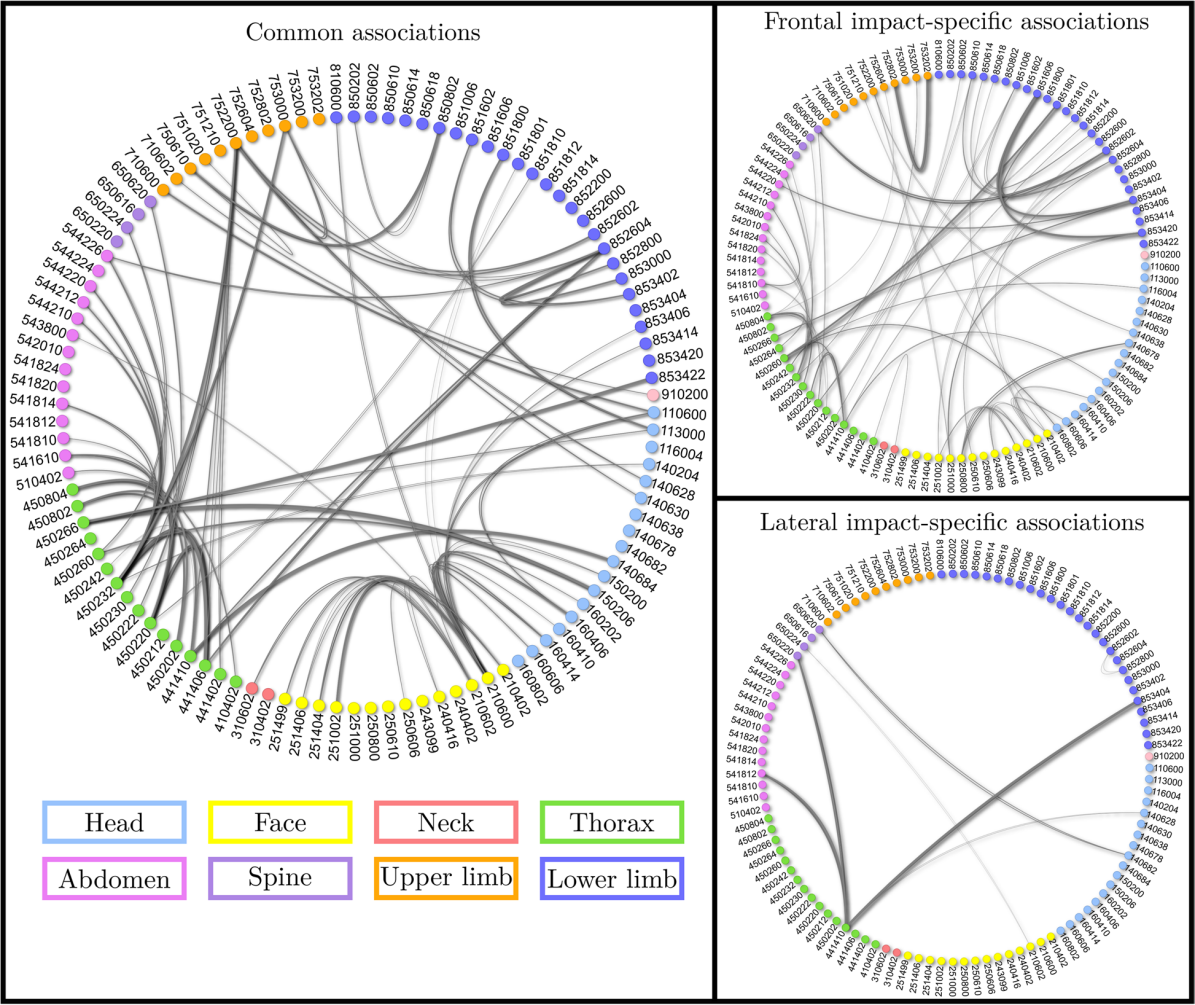
Injury associations using the RTSN classification. For an improved readability, all three subfigures are provided in higher quality as supplementary material. Subfigure 1: Common injury associations between frontal and lateral impacts. Subfigure 2: Injury associations specific to frontal impacts. Subfigure 3: Injury associations specific to lateral impacts

We also observed that the results obtained using the RTSN classification are rather different from the ones obtained using the BC., while they were computed from the same data. Notably, a higher number of associations was found for BC+AIS (Fig. 5) versus RTSN (Fig. 8). As introduced earlier, this is mostly attributable to the grouping of AIS codes which condense several injuries under the same BC label. This increases the prevalence, reduces the cardinality of the set of all studied injuries, and thus the number of parameters in the models, resulting in an improved statistical power to detect associations. One main strength of the present study is the complementarity of RTSN and BC analyses. The BC, by grouping several TSN and severity codes, unveils some associations that would not appear otherwise, such as several associations involving the spine that were not observed using the more precise RTSN classification. However, it prevents to evaluate association between unique injuries from the same type of tissue within the same body region. Without the RTSN classification, the identification of the clavicle fracture as possible index to help paramedics and doctors in their diagnosis and decision-making process would not have been feasible. This highlights the complexity of injury associations and patterns, and the pertinence of having a two-scale approach when studying injury associations: a global approach with the BC to point out the relationships between body structures, and a detailed approach (using the RTSN classification) allowing a precise identification of the wounds.

This study suffers from certain limitations. First, the PDOF was only described through 4 categories of accident type in our database, which may oversimplify the exact PDOF. It has been shown that oblique impacts have their own mechanisms and injury patterns [38], however this category does not exist in our database. As a consequence, oblique impacts might have been indifferently put in either “frontal” or “lateral” ones. A possible side-effect is that crashes with distinct consequences on the car occupants may have been put together in the same stratum, possibly reducing the between strata heterogeneity and thus artificially increasing the similarity of both frontal and lateral injury patterns.

Second, the AIS coding is directly related to the victim’s management and based on information available in medical records. Hence, because of the emergency of the management of road accident victims, clinicians might have overlooked minor lesions where more serious ones were present. This may have undermined the number of associations between severe and mild injuries, while some of them are of high interest, as it was explained earlier with the clavicle fracture. However, this possible bias was mitigated by the fact the coding was performed retrospectively by an ER physician based on the patient medical images and physiological analyses.

The study did not consider the most recent cases within the database (after 2014), and the classification used (AIS1990) is not the most recent version of the AIS. This might undermine the direct application of the results of this study for policy-making. However, this choice is due to two main reasons: the Rhône registry used the AIS1990 classification up until 2014, so the wide majority of the database entries are coded using this classification. Moreover, a direct translation to AIS1990 to more recent versions is not always possible and requires the patient medical records which are not available in the database. So, in order to keep the highest statistical power possible by having the largest number of cases possible, the choice was made to consider the cases before 2014.

Finally, the associations were computed only two-by-two. As shown in Ballout et al. [12], it is possible to consider interactions between injuries (second, third or higher-order interactions) to take into account the potential associations between three or more different injuries as well. However, the computational burden may rapidly become an issue due to the considerable increase of parameters in the models. Although in the BC we defined groups of injuries based on a priori knowledge (e.g., shared anatomic and tissue characteristics) contrary to models including interactions between injuries allowing to test all possible groupings for an exploratory purpose), using the BC is a way to reduce the number of groups of injuries to be tested and study associations between knowledge-defined groups of injuries at a lower computational price.

## Conclusion

The article presented an innovative approach to exploit an epidemiological database of road accident victim injuries. First, injury associations were computed on data stratified by accident type (lateral/frontal impacts). Then, an alternative injury classification was proposed aiming at better highlighting the biomechanics underlying injury associations. Finally, a thorough analysis of these associations was performed which led to the following main conclusions:

- The global association patterns are similar in frontal and lateral impact suggesting that most of them are injury-mechanism independent.
- Several impact-specific injury associations were found and could be linked to either particular injury mechanisms (e.g. contact with driving wheel) or differences in the human body mechanical properties between antero-posterior and fronto-lateral axes.
- Several impact-specific injuries were identified as being involved in a high number of associations suggesting that they are typical of polytraumatized victims.
- Several identified injuries are associated with blood vessels injuries that are known to require a fast management to optimize the victim’s chances of survival.

These last two points are of particular interest in a reduction of motor-vehicle-crash-related morbidity and mortality perspective. Indeed, they can orientate paramedics and doctors toward a faster and more appropriate decision. Table 8 presents two examples of how associations can be utilized to support on-field diagnosis.

**Table 8:** Examples of how injury associations can be utilized to support on-field diagnosis

Crash characteristics	Injury(ies) observed	Key associated injury(ies)
Frontal	Clavicle fracture	Increased risk of polytraumatism
		Increased risk of hemorrhage
Lateral	Costal fracture	Increased risk of polytraumatism
		Increased risk of hemorrhage

Future works shall include a broader number of accidents to be able to explore more injury mechanisms, especially the least represented. Moreover, this approach could be extended by including other categories of road user (pedestrian, two-wheeled, bike) and modifying the stratification accordingly. Overall, this exploratory work revealed the potential that injury association analyses have to inform policy-making and issue recommendations to decrease road accident mortality and morbidity.

## Data Availability

The datasets supporting the conclusions of this article are not publicly available. Data access requires a convention between the requesting party and Gustave Eiffel University and/or the French Road Safety delegation, subjected to a scientifically justified request guaranteeing the compliance with data access instructions. This data access request has also to be validated by the French data protection authority (CNIL).
